# Assessing Brazilian protected areas through social media: Insights from 10 years of public interest and engagement

**DOI:** 10.1371/journal.pone.0293581

**Published:** 2023-10-30

**Authors:** Carolina Neves Souza, João A. G. R. Almeida, Ricardo A. Correia, Richard J. Ladle, Adriana R. Carvalho, Ana C. M. Malhado

**Affiliations:** 1 Programa de pós-graduação em Diversidade Biológica e Conservação nos Trópicos, Instituto de Ciências Biológicas e da Saúde, Universidade Federal de Alagoas, Maceió, Alagoas, Brasil; 2 Instituto de Computação, Universidade Federal de Alagoas, Maceió, Alagoas, Brasil; 3 Department of Geosciences and Geography, Helsinki Lab of Interdisciplinary Conservation Science (HELICS), University of Helsinki, Helsinki, Finland; 4 Helsinki Institute of Sustainability Science (HELSUS), University of Helsinki, Helsinki, Finland; 5 Biodiversity Unit, University of Turku, Turku, Finland; 6 Centro de Investigação em Biodiversidade e Recursos Genéticos (CIBIO), Universidade do Porto, Vairão, Portugal; 7 Departamento de Ecologia, Universidade Federal do Rio Grande do Norte, Natal, Rio Grande do Norte, Brasil; Universidade Federal de Ouro Preto, BRAZIL

## Abstract

Social media platforms are a valuable source of data for investigating cultural and political trends related to public interest in nature and conservation. Here, we use the micro-blogging social network Twitter to explore trends in public interest in Brazilian protected areas (PAs). We identified ~400,000 Portuguese language tweets pertaining to all categories of Brazilian PAs over a ten-year period (1 January 2011–31 December 2020). We analysed the content of these tweets and calculated metrics of user engagement (likes and retweets) to uncover patterns and drivers of public interest in Brazilian PAs. Our results indicate that users / tweets mentioning PAs remained stable throughout the sample period. However, engagement with tweets grew steeply, particularly from 2018 onward and coinciding with a change in the Brazilian federal government. Furthermore, public interest was not evenly distributed across PAs; while national parks were the subject of the most tweets, mainly related to tourism activities, tweets related to conflicts among park users and managers were more likely to engage Twitter users. Our study highlights that automatic or semi-automatic monitoring of social media content and engagement has great potential as an early warning system to identify emerging conflicts and to generate data and metrics to support PA policy, governance and management.

## Introduction

Protected areas (PAs) are a key tool for biodiversity conservation [1]. In Brazil, these areas are not only responsible for protecting different ecosystems, habitats and endangered species, they also safeguard important cultural and socioeconomic values [2]. To align social and economic aims, in addition to conservation and recreation, the Brazilian system includes two broad categories of PAs: strictly protected and sustainable use. Indigenous lands also provide an important contribution to biodiversity conservation, although they are not formally recognized as PAs in the National Protected Areas System (SNUC) [3] and are regulated by different legislation [4]. The Brazilian PA system is one of the largest in the world, but is facing a range of challenges that threaten its integrity and long-term sustainability. Among the most pressing of these challenges are: i) its reliance on outdated top-down governance systems that do not sufficiently allow for the direct participation of society [5]; ii) the long term and persistent institutional crisis facing the federal biodiversity conservation agency [6]; iii) a lack of transparency in management actions and in communicating the importance of these areas, which tends to result in increasing environmental crimes and corresponding diminishment of management effectiveness and monitoring of these areas [7], and; iv) a growing funding deficit, which weakens PAs that are not able to cover their management costs [8]. In summary, fostering a stronger connection between Brazilian PAs and society is crucial to avoid them being perceived as opportunity costs by citizens and politicians [9]. In addition, understanding how people interact with these PAs can provide important insights on how to increase society’s support for conservation efforts.

Individuals interact with PAs in a wide range of ways, generating diverse values and evoking different interests and feelings [10]. In Brazil, depending on the PA category, citizens and visitors can engage in a wide range of activities including recreation, research, developing environmental education activities, or simply visiting and enjoying the iconic landscapes and biological spectacles. Such interactions have demonstrable psychological and physical benefits to humans and can promote well-being [11]. Nevertheless, Brazilian PAs are primarily configured for environmental conservation, with varying use restrictions that can contribute to a lack of interest from wider society and a general alienation from nature [12]. Given that public support is critical for the legitimacy of PAs [7], understanding human interactions, sentiment and public interest in PAs is essential for developing effective strategies to attract societal support and for supporting decision-makers and researchers in conservation planning, financing and public communication activities [13–15].

Traditionally, human interactions with nature have been investigated through social surveys which are necessarily costly and limited in scale. It has recently been suggested that the huge volumes of data generated by social media and other digital platforms could be a complementary approach utilised to quantify these interactions at a larger scale of population and geographic accessibility [16]—this field of study is called conservation culturomics [14]. In comparison to questionnaires, social media analysis has the potential to generate large amounts of data, at a lower financial cost, and on a larger geographical scale [13]. This in no way invalidates the continued use of questionnaires as a valuable methodological tool; data generated from analysis of social networks have many intrinsic biases [17] and, critically, does not capture the attitudes and behaviours of communities/individuals with limited access to the internet and/or those that do not use social networks [12, 18]. Another concern is the reliability of how the data is recorded and made available by the system. According to [19], inconsistent data measurements by the system can also undermine internal validity, making it difficult to infer causality from the responses. The authors therefore suggest that researchers familiarise themselves with the system used in the survey in order to validate the results. Although about 90% of Brazilians have access to the Internet, this demographic representation does not cover the entire country. Nevertheless, this limitation does not invalidate the usefulness of this investigative approach, since the results obtained in the research may often have relevance beyond the scope of social network users [18]. Culturomic data is unrivalled in its potential for systematically capturing, identifying and mapping human-nature interactions at large spatial and temporal scales [17]. Indeed, social media data has already been used successfully to inform science communication [20, 21], investigate ecotourism in high conflict environments [22], assess online sentiment towards threatened species [23], enhance public awareness of wildlife conservation [24], and to better understand public perceptions and feelings related to PAs [12, 13, 25]. In Brazil, Google Trends data has previously been used to assess public interest and internet salience in relation to Brazilian PAs [26]. However, more studies are needed to assess the relationship between social media content and PAs, as well as to investigate public sentiment towards these areas.

Here, we use data from the social media platform Twitter (recently renamed X) to investigate public attitudes and interest in Brazilian PAs. Twitter is one of the most popular social media and microblogging platforms with over 436 million active users worldwide in 2021 [27]. In Brazil, Twitter has up to 14.1 million active users, who posted millions of comments (so-called “tweets”) every day containing thoughts and opinions of up to 280 characters [28]. Despite the character limit, the inclusion of links significantly enhances the informational content and potential impact of tweets, as it enables individuals to access additional resources, broaden their knowledge, and engage in more enriching and informed discussions. The company has promoted itself as the right place to learn more about “what’s going on” and “what people are talking about right now”. Twitter is heavily used by journalists, scientists, politicians, managers, and wider society [29, 30] to spread information, promote public discourse, and thus serves as a potentially sensitive barometer of public opinion [31]. However, it is important to note that due to its open nature Twitter can also be responsible for the dissemination of misinformation and the spread of fake news.

In this study we analysed ten years (2011–2020) of public tweets in Portuguese language that contained content related to Brazilian PAs with the objective of answering the following questions: (i) what is the volume of posts on Twitter about Brazilian PAs? (ii) where are people communicating about Brazilian PAs? (iii) which types of Brazilian PAs generate more posts and engagement (e.g., other users reacting to the original posts through ‘likes’ and ‘retweets’)? (iv) what is the relationship between the number of posts and the engagement of users who post about PAs? (v) what are the most discussed topics in the posts? Answering these questions through digital data analytics can inform targeted communication strategies, improve public engagement and foster a deeper understanding of biodiversity conservation, leading to more effective and impactful conservation initiatives.

## Material and methods

### Brazilian protected areas

Brazil has enormous biodiversity and an extensive system of conservation units, protected spaces that are part of the Brazilian territory and that are managed to conserve its ecological, historical, geological, and cultural heritage [32]. Since Brazil committed to international programmes such as the Convention on Biological Diversity (CBD) and national targets aimed at conservation, the country’s PAs system has rapidly expanded. Although the system of PAs in Brazil was not directly created in response to the CBD, the Convention served as a backdrop for the establishment of the National System of PAs (SNUC). (This occurred because the initial versions of the law that instituted the SNUC predated the CBD). During its passage through the National Congress, which took approximately 12 years, some of the guidelines from the Convention were incorporated into the text of this legal framework, thereby making it the primary instrument focused on biodiversity conservation in Brazil [33]. The consolidation of the various norms regarding Conservation Units in Brazil was not straightforward due to frequent disagreements between conservationist and preservationist perceptions of these areas [34]. However, after a long process of discussions among technicians, researchers, and public bodies, the National System of Conservation Units (Sistema Nacional de Unidades de Conservação, commonly referred to by its acronym SNUC) was unified under a single law (see [3]).

The SNUC recognizes 12 categories of conservation units, separated according to their management objectives and types of use. These categories fall into two major groups: strict protection and sustainable use PAs [3]. Most, but not all, of Brazil’s PAs are documented in the National Registry of Protected Areas (Cadastro Nacional de Unidades de Conservação—CNUC). The CNUC currently includes 2,659 PAs, including marine and private PAs, managed at federal, state, and municipal levels. These areas cover about 18.80% of the continental area and 26.48% of the marine area of Brazil [35]. Between 2003 and 2009, Brazil was solely responsible for 74% of the global increase in PA coverage (km^2^), mainly due to several large PAs created in Amazonia [36]. Despite Brazil’s leading role in global conservation and the immense success of its PA programme, Brazilian politicians and decision-makers seem to be increasingly viewing PAs as opportunity costs that limit economic development, leaving many PAs vulnerable to downgrading, downsizing or degazettement (PADDD) [37]. In this context, demonstrating public support for PAs and revealing their true value to society is an essential step to ensure their long-term sustainability [9].

### Data collection

**Fig 1:**
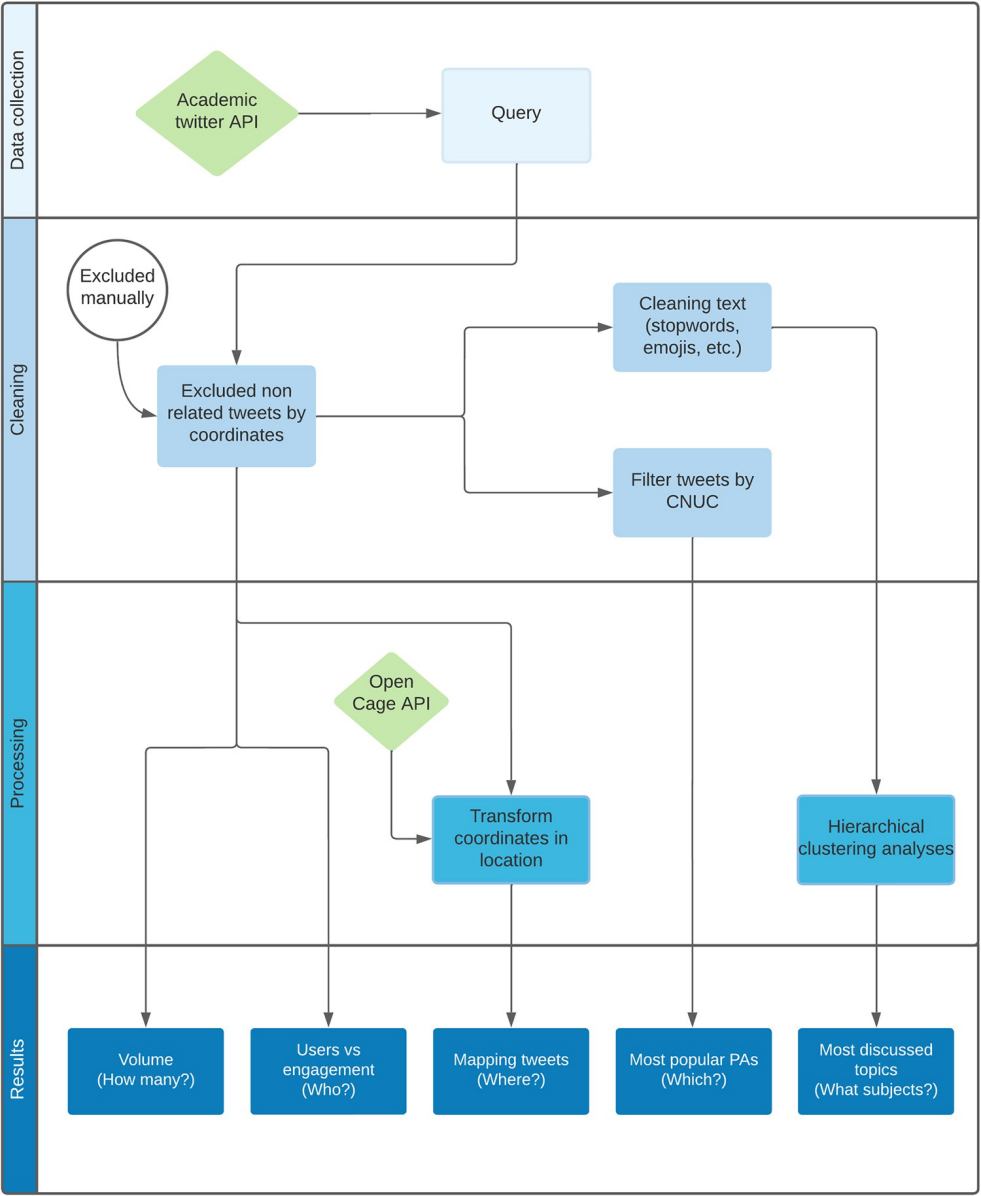
Methodological flowchart. Methodological flowchart from data collection to results. The flowchart shows all the steps used during the research: data collection, cleaning, processing, and analysis.

Digital data for conservation culturomics analysis can be collected from different sources (e.g., texts, videos, images, songs) [38]. Based on the framework suggested by [17], we analysed the content of, engagement with and author characteristics of publicly available Twitter posts about Brazilian PAs in Portuguese language. The data mining techniques involved data collection, cleaning, processing and analysis (see Fig 1). In this study, we utilised the Twitter v2 API to collect all the data. Twitter’s Academic access to its v2 API has provided us with the opportunity to gather up to 10 million tweets per month, which is a significant increase of 20 times compared to what was previously possible with the standard v1.1 API [39]. Moreover, this access allows us to retrieve older conversation histories, making Twitter a rich and accessible source of data for textual content analysis. As such, it becomes a valuable tool for gaining insights and a better understanding of public discussions and perceptions about Brazilian PAs. The code for data mining was developed using the Python language program v.3.9 (http://www.python.org) and was based on the Full-Archive-Search API node example from Twitter’s official repository.

The second step was to define which content we would like to collect from Twitter. A query of 18 keywords was defined in the Portuguese languaged to extract the tweets (not the retweets).: 1- Parque nacional (National park); 2- Parque Estadual (State park); 3- Parque natural municipal (Natural municipal park); 4- Parque municipal (Municipal park); 5- Estação Ecológica (Ecological station); 6- Reserva Biológica (Biological reserve); 7- Monumento Natural (Natural monument); 8- Refúgio da Vida Silvestre (Wildlife refuge); 9- Reserva Extrativista (Extractivism reserve); 10- Área de proteção ambiental (Environmental protected area); 11- Floresta nacional (National forest); 12- Floresta estadual (State forest); 13- Floresta municipal (Municipal forest); 14- Reserva de desenvolvimento sustentável (Sustainable development reserve); 15- Área de interesse relevante (Area of relevant interest); 16- Reserva particular do patrimônio natural (Private natural reserve); 17- Unidade de conservação (Conservation unit); 18- Área protegida (Protected area). All 12 categories of Brazilian PAs were included in the query. PAs with management levels in their name were also added to the query, for example, “national park”, “state park”, and “municipal park”. Furthermore, to collect the tweets that do not mention the names of PAs in their textual content, the query also included the two keywords: conservation unit and protected area (unidade de conservação e área protegida). We also considered adding to the set of terms used in the query the acronyms referring to each PA category, for example APA for Área de Proteção Ambiental (Environmental Protection Area). However, a preliminary analysis of data collected using the acronyms revealed these mostly relate to other topics such as slang, celebrity names and other words that did not result in tweets discussing PAs. Based on this, even though it resulted in reducing our sample size, we decided not to include the acronyms in the query to reduce bias and noise. The collection of the textual content of retweets was also avoided to prevent possible biases in the results of the multiple counting, and in the salience of topics, since they generally represent repetitive copies of original tweets. Instead of collecting the textual content of retweets, we collected quantitative data from the original comments that contained the number of times the message was retweeted, received likes or comments. In addition, the use of keywords in our query made it possible to identify and collect all messages, including retweets that had comments related to the original tweet, that mentioned any Brazilian PAs. The tweets were retrieved from the API between April 15th and April 30th, 2021. The sampling period encompassed tweets posted from January 1st, 2011, to December 31st, 2020. In total, before the data cleaning and filtering process, 421,254 tweets were collected.

The following information was collected from each tweet: (i) author (name, username, and whether it is verified); (ii) date of publication; (iii) geographical data (latitude, longitude, city, country); (iv) publication data (number of likes, retweets, replies and whether it is a reply to another user); and (v) the text of the tweet. The tweets were downloaded in JSON format, by year, with a maximum of 500 tweets per page (as per the limit set by the Twitter API). After converting the JSON pages to a single CSV file, the data cleaning process was performed. Initially, a filter was applied to the geographical metadata provided by Twitter (country and country_code columns) to identify tweets from countries other than Brazil. Subsequently, a manual verification of each foreign tweet was conducted to eliminate those that did not correspond to Brazilian PAs. Our final validated list contained a total of 402,508 tweets about Brazilian PAs (see all IDs collected from Twitter in https://github.com/jagra26/Brazilian-PAs-on-twitter). Tweets usually contain URLs, emojis, and emoticons, so the dataset needs to be cleaned before analysis. Text cleaning was performed using the R language program (R Core Team, 2017), with the tm package [40]. Specifically, we used the function ‘tm_map’ to convert all text to lower case and remove any hashtags, URLs, symbols (like □, ■, ◆), numbers, and Portuguese stop words present in the text. In terms of data collection, it’s worth noting that the availability of the APIs for academic usage has been volatile recently, as the platform is now referred to as X and has undergone a policy change in data access which has restricted access.

### Data analysis

We aimed to understand the public interest in Brazilian PAs based on the number of tweets and public engagement with those posts. First, we compared the volume of tweets about Brazilian PAs on a temporal scale of 2011 to 2020 with the number of users who post about PAs. To do this, we counted the number of tweets, number of unique users, and the total number of ‘likes’ and retweets by year to create a line graph. To explore the relationship between the volume of tweets and engagement, we summarised the number of tweets and the average number of ‘likes’ and retweets per post at the user level. We calculated the bootstrapped mean engagement over time using 1000 samples and a confidence interval of 95% with function ‘smean.cl.boot’ from R package Hmisc [41], and generated a line plot depicting the disparity between user types (verified and not verified). We also bootstrapped the Spearman’s correlation between number of tweets and mean engagement per tweet using 1000 samples and 95% confidence intervals with function ‘spearmanRho’ from R package rcompanion [42].

We also mapped the number of Tweets about Brazilian PAs based on location information provided by Twitter’s users. To identify the geolocation of tweets related to Brazilian PAs, we employed a keyword-based (see query used in the data collection section) sampling strategy that increased the likelihood of capturing tweets about the targeted PAs originating from various locations [25]. The metadata of the tweets provided geographic data such as coordinates, countries, and cities. However, the consistency of this information was limited, with many tweets containing only a single geographic coordinate (e.g., the absence of country-level data despite the availability of coordinates). To overcome this limitation, we adopted a similar approach to [43], where we utilised the OpenCage Geocoding API search engine (https://opencagedata.com) for reverse geocoding. This process allowed us to obtain comprehensive state, and country information, based on the coordinate metadata provided by Twitter. Ultimately, our geospatial dataset consisted of 62,924 tweets (15.63%) with location data (geographic coordinates and states and countries data). Using the folium library [44] in Python version 3.9 (http://www.python.org), we created a choropleth map where the colour intensity of each territory corresponded to the number of tweets posted.

It was necessary to implement a filtering process to assess which Brazilian PAs had the most posts, as the majority of tweets did not explicitly mention the full names of the PAs in their text content. To accomplish this, we filtered our dataset using the proper names of the PAs from the National Register of Protected Areas (MMA/CNUC 2021), without considering the specific category of the PA in the search, using the VLOOKUP function through Microsoft Excel software (Microsoft 365 2020): = VLOOKUP (search_value, SEARCH (search_text, no_text, [start_num]), search_text). Exceptions to the filtering process using the full name with the category type were made for those areas that had the same name with different categories, such as "Tamoios Ecological Station’’ and "Tamoios Environmental Protection Area." The list of names used for filtering can be accessed on the repository (https://github.com/jagra26/Brazilian-PAs-on-twitter). The final dataset used in this analysis has 189,294 tweets containing PAs in CNUC. Using the new dataset, we generated a lollipop chart using summarised data on the number of posts and average engagement per PA to compare the differences between the public interest of each area.

Finally, to group tweets based on similarity in content and to identify the main topics Twitter users discuss concerning Brazilian PAs, we applied the agglomerative clustering technique, known as AGNES (Agglomerative Nesting). The clustering method used to calculate the distances was the "complete" method, based on the word composition of posts in the dataset [45]. The complete method uses the “largest dissimilarity between a point in the first cluster and a point in the second cluster (furthest neighbour method)” [46]. According to the study conducted by [47], in order to ensure that the final clusters became neither excessively broad (involving few clusters covering PAs that share the most common words) nor excessively specific (resulting in numerous clusters addressing PAs that share less frequent words), the authors carried out several simulations with a range of 2 to 10 clusters (k). During these simulations, the most frequent words in each cluster were examined in each scenario in order to identify the most prominent characteristics of each cluster in relation to the Brazilian PAs. The cluster analysis produced a dendrogram in which the distances between the branches reflect the similarity between each tweet. This procedure made it possible to define the theme of each cluster (5) based on the similarity of the words grouped in the dendrogram. With exception of the choropleth map, all analyses were performed in the R programming language. The following packages were used in the analysis: dplyr package was used for the data manipulation [48]; to create the graphics we used the packages ggplot2 [49] and plotly [50]; and, finally, for the hierarchical clustering analysis, we used the packages cluster [45] and factoextra [51]. All R and Python scripts used for data collection and analysis are available on our repository page (https://github.com/jagra26/Brazilian-PAs-on-twitter).

## Results

### Volume of tweets about Brazilian PAs

**Fig 2:**
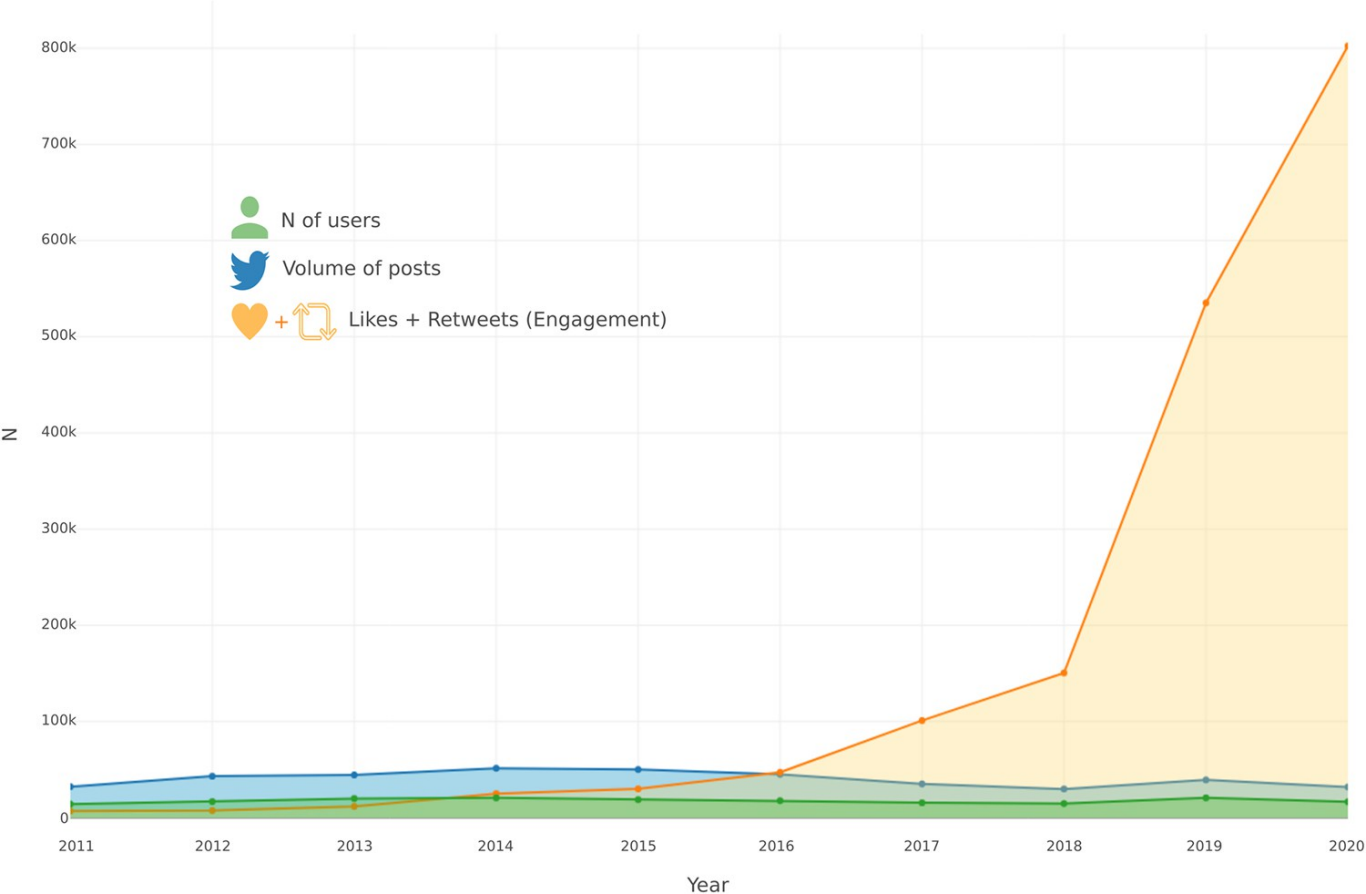
Volume of posts, users and engagement of Brazilian protected areas. Line graph representing the volume of users, tweets, and public engagement (likes + retweets) about Brazilian protected areas during the period from 01 January 2011 to 31 December 2020.

**Fig 3:**
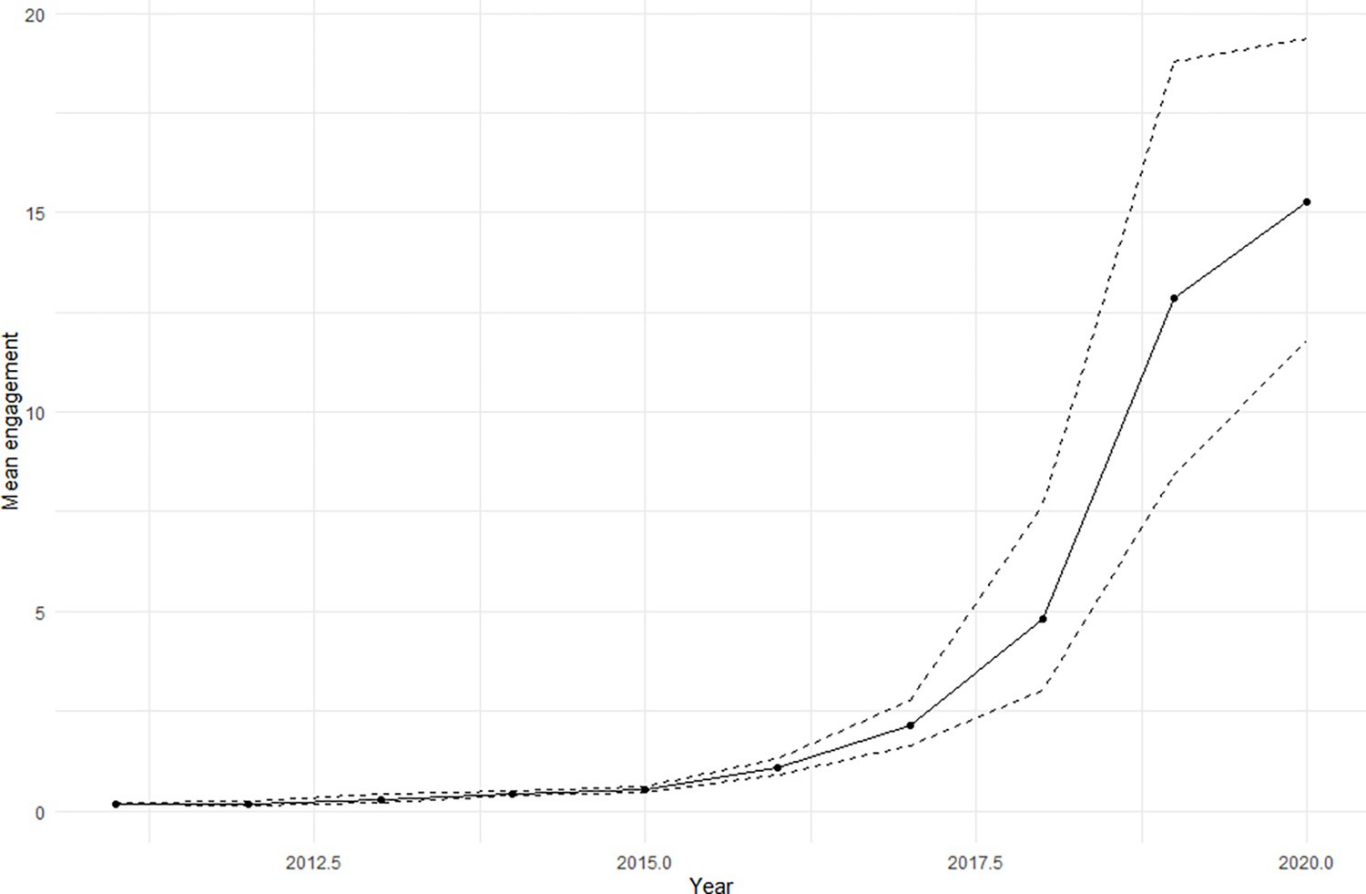
Bootstrapped estimates of mean post engagement per year. Figure shows the results of a Bootstrap analysis representing the mean engagement (likes and retweets) with posts about Brazilian protected areas published between 2011 and 2020.

**Fig 4:**
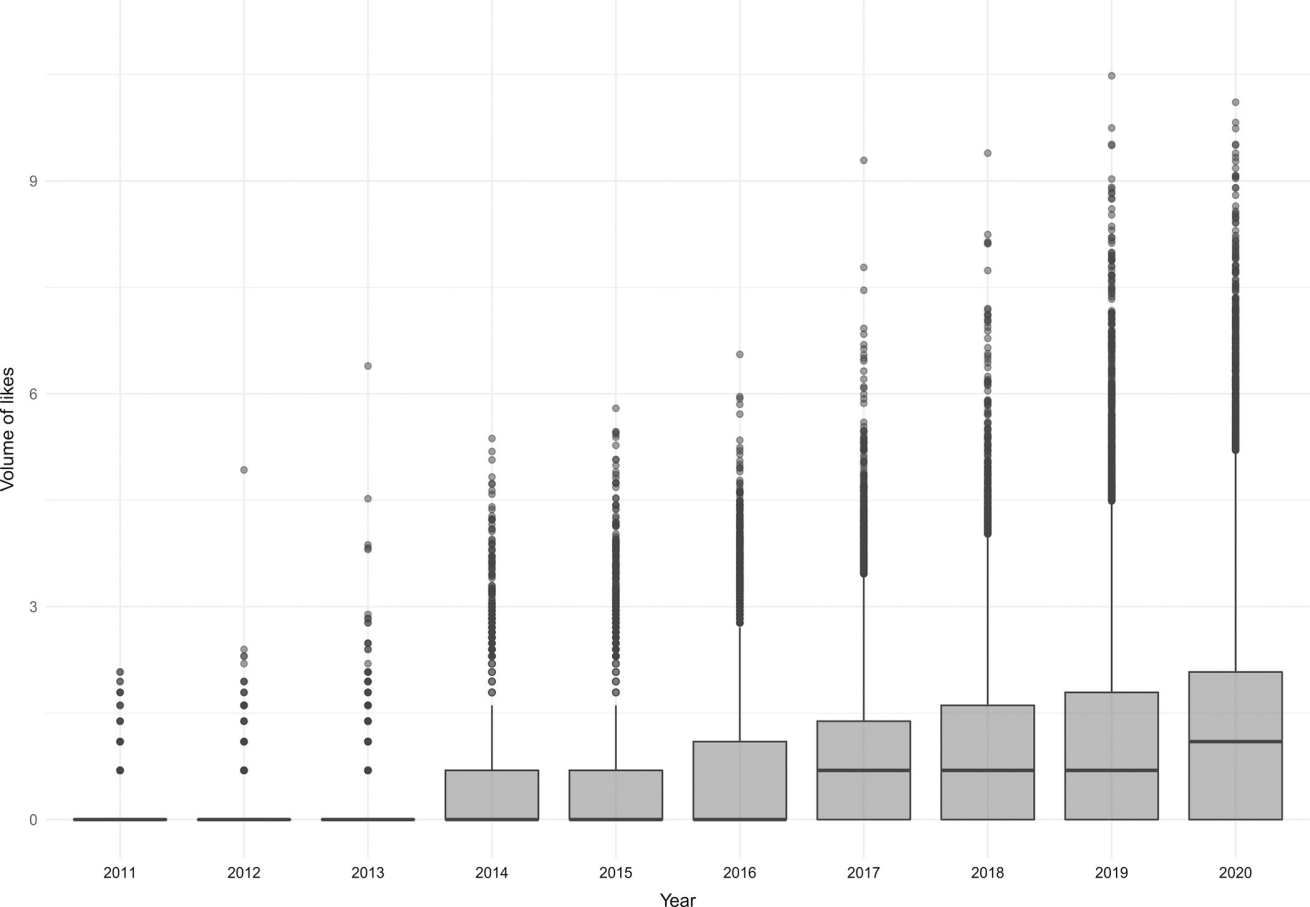
Public interest related to the Brazilian protected areas over the years. Boxplot represents the distribution of the volume of likes for Brazilian protected areas during the period from 01 January 2011 to 31 December 2020. The lower and upper box limits correspond to the first and third quartiles (the 25th and 75th percentiles); a black bar inside the box indicates the median. The volume of tweets was log-transformed (natural logarithm).

We mined a total of 402,508 valid tweets posts about Brazilian PAs between 2011 and 2020, which were those that successfully passed through the data cleaning process. The number of posts and the number of users tweeting about the topic was relatively similar throughout the decade analysed, with only minor decreases in the number of posts after 2016 (Fig 2). The average number of posts and users posting about PAs in Brazil was around 40,000 posts and around 17,000 users over the 10 years analysed. Metrics of engagement (likes + retweets) with posts about PAs started increasing in 2016, but grew steeply after 2018. Estimates of the mean engagement per tweet increased from 0.182 (bootstraped 95% C.I.: 0.165–0.199) in 2011 to 15.3 (bootstrapped 95% C.I.: 11.9–19.6) (Fig 3). Compared to 2018, the number of people actively posting about PAs in Brazil in 2019 increased by 40%, posts about what is happening in these areas increased by 31%, and public participation increased by 255% (Fig 4).

### User characteristics

**Fig 5:**
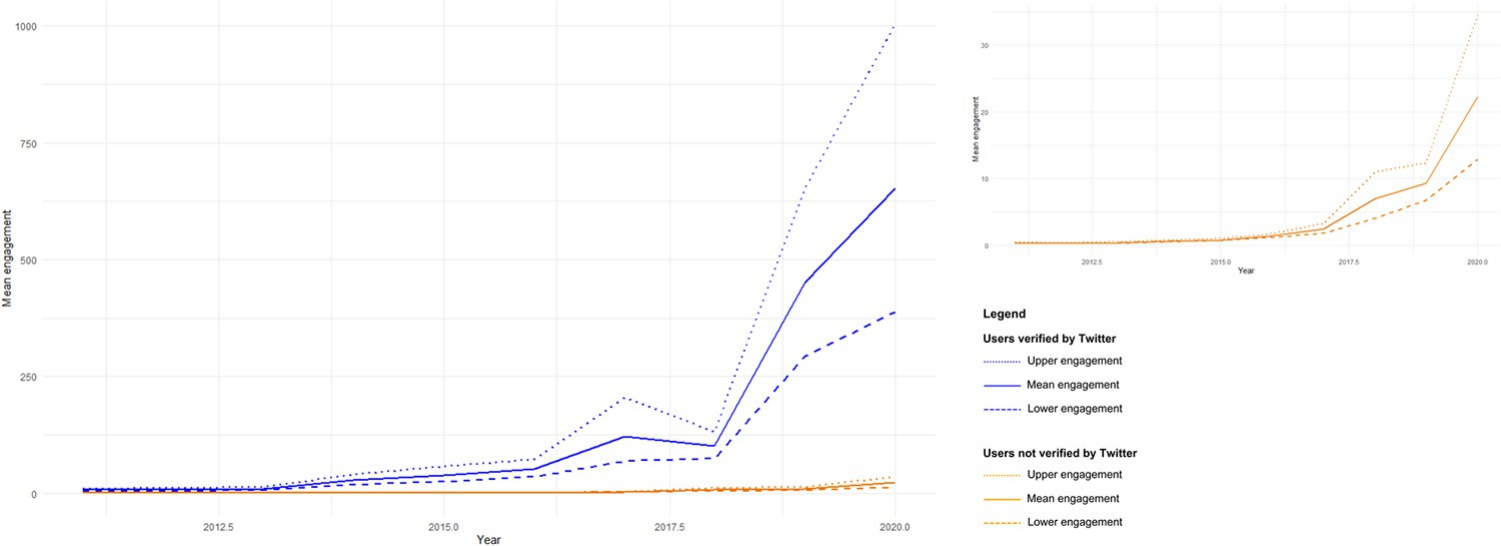
Bootstrapped estimates of mean post engagement per year and per user type. Figure shows the results of a Bootstrap analysis representing the mean engagement (likes and retweets) with posts about Brazilian protected areas, per type of user (verified and not verified) published between 2011 and 2020.

We identified 130,742 Twitter users who have posted content about Brazilian PAs. Some of these Twitter users have many followers, including news media, politicians, celebrities, travel agencies, and a range of international people and organisations such as WWF and UNESCO. These are frequently classified by Twitter as ‘users of public interest’ and, in most cases, received a verification seal. We explored the relationship between user type (verified or not) and the number of tweets about Brazilian PAs and the public interest in those posts (Fig 5). In general, users who post more about Brazilian PAs receive more engagement (likes + retweets). Indeed, there was a small but positive correlation (Spearman’s rho = 0.107; bootstrapped 95% C.I.: 0.102–0.112) between the number of tweets per user and mean engagement per tweet. However, it is worth noting that when we compared engagement between verified and not verified users, our bootstrap analysis showed that the estimates of the average engagement per tweet of an not verified user increased from 0.354 (bootstrapped 95% CI: 0. 305–0.409) in 2011 to 22.3 (bootstrapped 95% CI: 12.9–34.5) in 2020, while the average engagement per tweet of verified users increased from 7.00 (bootstrapped 95% CI: 4.48–10.1) in 2011 to 651.8 (bootstrapped 95% CI: 388.6–1003.9) (Fig 5). For example, of the 10 users who have posts with an average engagement of more than 5,000 likes and retweets, seven are verified profiles and have a considerable number of followers (>15,000 followers). Interestingly, the most of these users, despite their large following, posted only one or two tweets related to Brazilian PAs during the analysed ten-year period.

### Geographic focus of tweets

**Fig 6:**
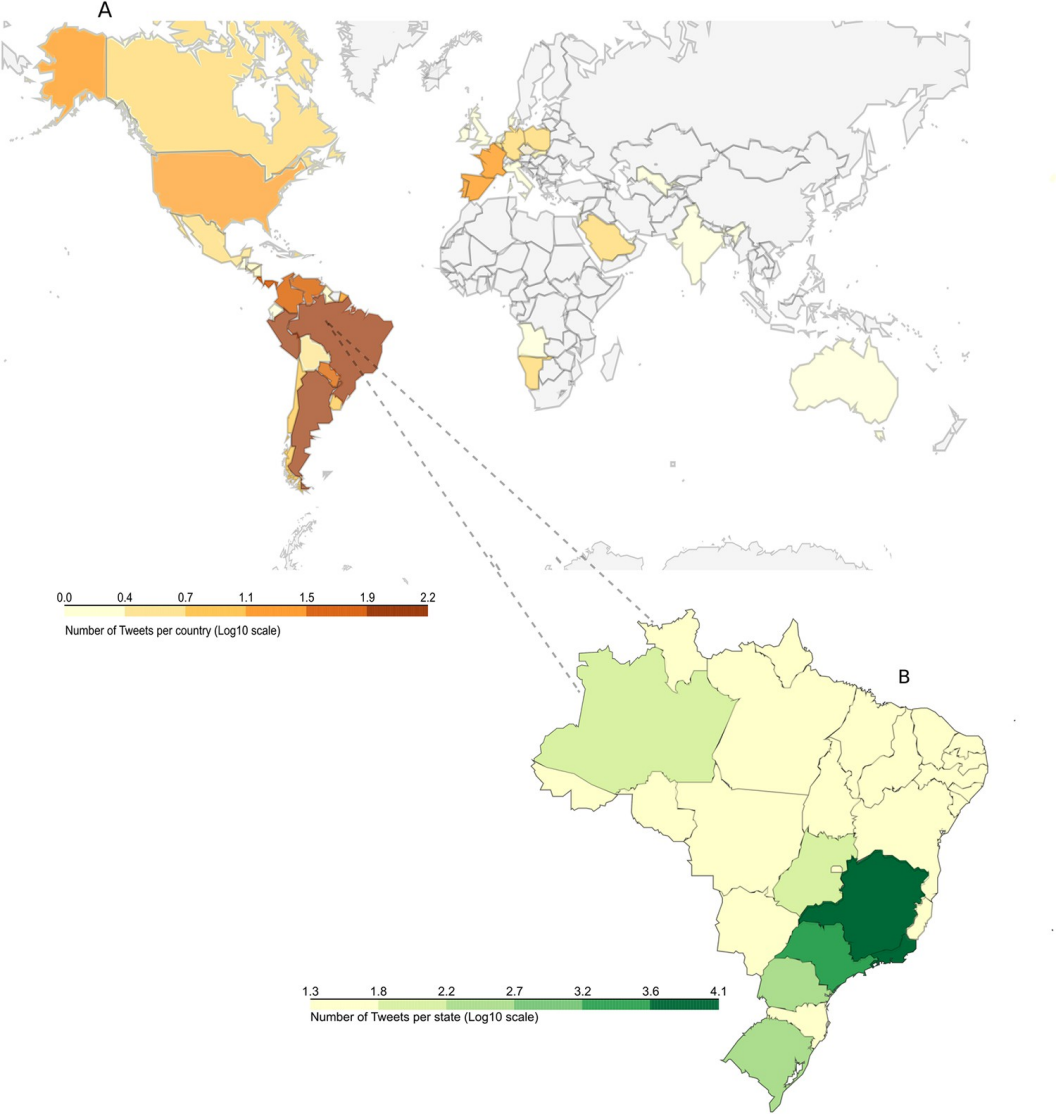
Geographical distribution of the tweets posted about Brazilian protected areas. Choropleth map representing the number of tweets posted about Brazilian protected areas in Brazil and worldwide from 2011–2020. Locations are extracted from geotagged tweets and Twitter user profiles. The map was cropped above Alaska to enhance the visibility of the other countries.

The large majority of tweets (provided by the Twitter coordinates metadata) about Brazilian PAs originate in Brazil, as expected, but we also found mentions from countries bordering Brazil, Costa Rica, Portugal, and the United States (Fig 6). This result is similar to the spatial distribution found by lusophone and associated countries (https://www.cplp.org/*)*. In Brazil, the state of Rio de Janeiro received the highest number of georeferenced tweets about PAs (12,585), followed by Minas Gerais (11,339), São Paulo (8,837), and Paraná (5,434). Nevertheless, when we examine which PAs were most georeferenced by Twitter users, we can see that out of the top 100 geo-referenced areas, 64% are urban parks. The most geotagged National Park was Iguaçu National Park (Paraná state) with 2,747 tweets, followed by Tijuca National Park (Rio de Janeiro state) with 1,300 tweets and Serra dos Órgãos National Park (Rio de Janeiro state) with 752 tweets. The most geotagged urban park was Américo Renné Giannetti Municipal Park (Minas Gerais state) with 2,615 tweets, followed by Ponte dos Bilhares municipal Park (Manaus state) with 1,101 tweets and Flamboyant Municipal Park (Goiás state) with 1,033 tweets.

### Which protected areas generate most public interest and engagement?

**Fig 7:**
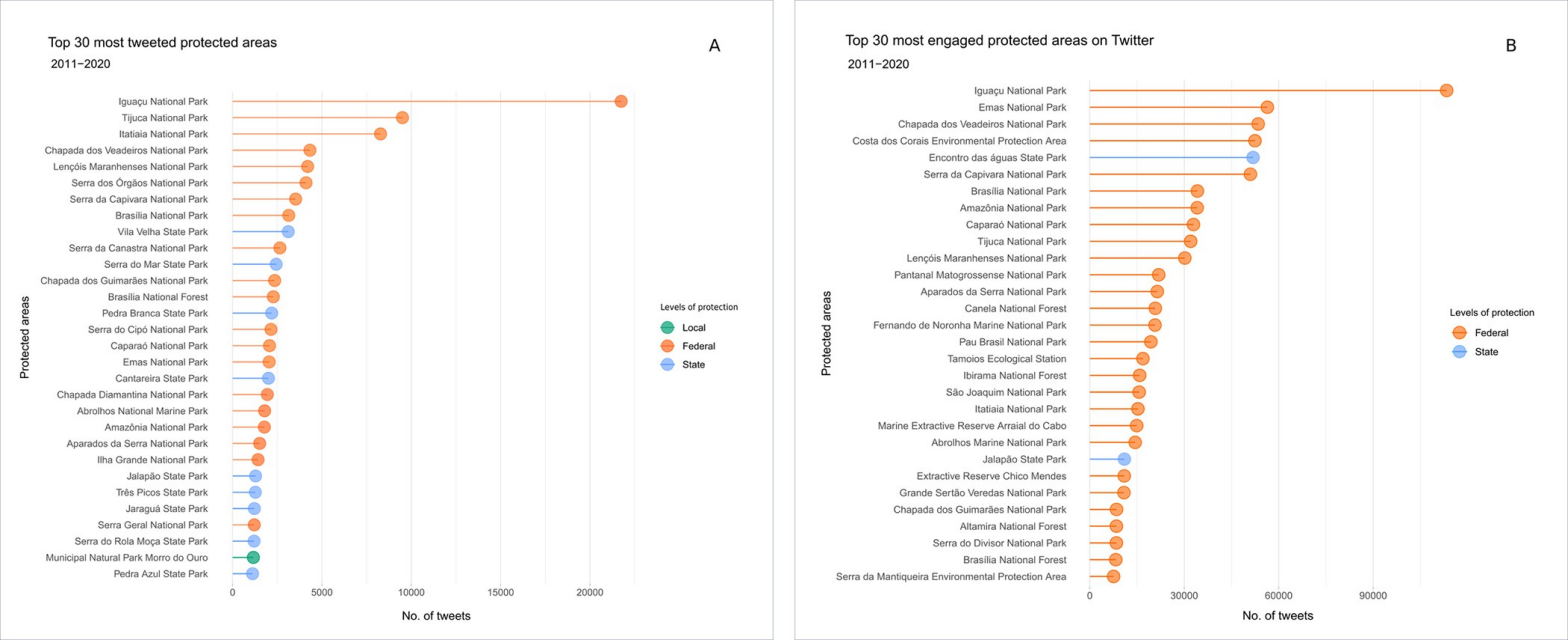
Brazilian protected areas that have generated the most public interest and engagement. (A) Lollipop plot illustrating the volume of tweets about Brazilian protected areas during the period from 01 January 2011 to 31 December 2020. (B) Lollipop plot illustrating the average of engagement in the tweets related to Brazilian protected areas. The top 30 tweets were illustrated in ascending order and grouped by level of protection: federal, state, and local.

Based on the analysis of tweet content, national parks were the most posted category of PAs on Twitter, followed by state and municipal parks (Fig 7A). The iconic Iguaçu National Park received over 21,700 tweets, and Tijuca National Park received 9,507 tweets. Tourism emerged as the primary topic tweeted about among the top 30 most-tweeted parks; seven out of the ten most visited national parks in Brazil [52] were among the top 12 most-tweeted about parks.

We hypothesized that PAs that generated high public interest, with numerous posts about them, also garnered high engagement (likes + retweets). When evaluating the average engagement associated with PAs, besides national parks, other PA categories surfaced, including environmental protection areas, national forests, and biological reserves (Fig 7B). In addition to Iguaçu National Park, our results indicate that the most tweeted PAs did not achieve the highest levels of engagement (see comparison in Fig 7A and 7B). Instead, tweets about conflicts, rather than tourism activities, generated the highest engagement, and these tweets were more frequently associated with other PA designations. Upon analysing the five most engaged PAs on Twitter, the tweets with the highest engagement included discussions about fires, jaguar deaths, and administrative abuses, among the most frequent topics.

### Content analysis of tweets

**Fig 8:**
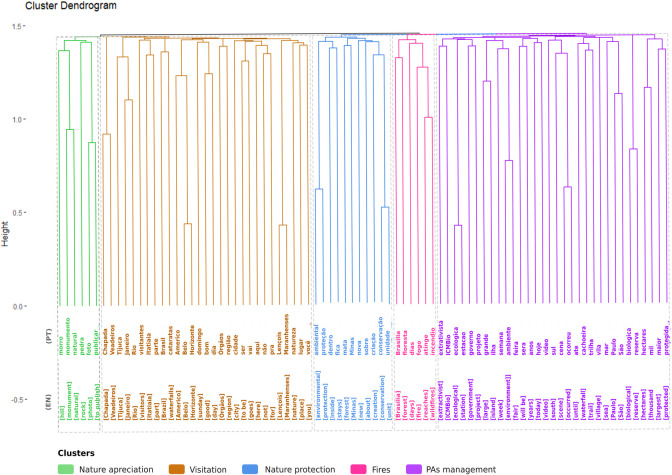
Most published content related to Brazilian protected areas. The contents were published in the period 01 January 2011 to 31 December 2020. Each branch in the dendrogram represents a different cluster, grouped according to the similarity of words and themes.

A broader look at the topics discussed in the full dataset revealed somewhat similar patterns. Our cluster analysis of the most commonly featured keywords in all tweets identified five thematic categories. Based on the most frequent words in each cluster represented in the dendrogram (Fig 8), we characterised the main topics of discussion as follows:

- Fires: this cluster included words associated with fires in PAs;
- Management of protected areas: themes related to environmental crimes; PADDD (downgrading, downsizing, and degazettement) events, mainly focused on the size of PAs, and educational campaigns;
- Nature protection: with regard to the creation of new PAs;
- Nature appreciation: this cluster included words associated with sharing on social media and natural monuments;
- Visitation: where the names of national parks, and urban parks were mentioned the most.

## Discussion

We assessed Twitter users’ interest in Brazilian PAs over a ten-year time scale (2011–2020) using metrics of content and engagement, including likes and retweets in the Portuguese language. Our results suggest that social media content related to PAs remained broadly stable throughout the studied period, though engagement with this content was low until 2016, at which time engagement began to increase. Engagement grew particularly steeply after 2018, following a remarkable increase in likes and retweets related to Brazilian PAs, even though the number of users posting on this theme remained relatively stable over time. There are at least two factors that could be driving this pattern: First, in 2016 Twitter introduced an algorithm to customise the content that users can access on their profile’s timeline [53]. Based on accounts users have chosen to follow and posts interacted with, the algorithm presents users with a series of recommendations. This new personalization algorithm amplifies certain content while reducing the visibility of other posts [54]. In this way, people sympathetic to environmental themes will be more likely to be presented with content related to nature (an “echo chamber” effect), such as PAs, and may more readily engage with posts (tweets and retweets) that in the past may not have been easily encountered. In addition, users who carried the verification seal on Twitter, identifying them as figures of public interest, may have experienced a significant increase in visibility and influence within the platform after 2016 (Fig 5). This change can be attributed to the way Twitter’s algorithm has optimised the dissemination of tweets from these verified users, especially by making their tweets become viral.

A second reason for the observed engagement trend may be related to the change in Brazilian federal government that took place at the end of the same year, and the increase in public discourse about the environment and environmental policy that started during the political campaign that preceded the election and continued after it took place. In 2019, the recently elected Bolsonaro government made a number of highly controversial decisions to backtrack on environmental policies [55]. Specifically, the incoming government made pledges to halt the expansion of the PA system and to make environmental licensing more flexible [56] leading to a wide scale mobilisation of the environmental movement in Brazil. The resulting conflicts of perspectives and attitudes generated considerable discussion on Twitter and beyond. This, combined with Twitter’s new algorithm, may have prompted concerned citizens to express their opinions (agreeing or disagreeing with government policy) by engaging with content related to what was happening to Brazilian PAs.

Notwithstanding the highly polarised debate around Brazilian PAs and improvements in personalization of Twitter feeds, our results indicate that the volume of posts made by a single user was positively but weakly correlated with levels of engagement. Also, posts by environmental NGOs, official agencies and celebrities had a much greater level of engagement, as has been found in other studies [25]. As illustrated in Fig 5, the growth trajectory of engagement regarding PAs after 2016 is similar between the types of users (verified and unverified). Nevertheless, it is verified users who play a key role in feeding interest and engagement in this subject, due to the substantial number of followers, running into thousands or even millions, who often express appreciation through likes, comments and retweets—a result which, perhaps unsurprisingly, highlights the ability of celebrities to captivate the Twittersphere’s attention. Celebrities have the ability to attract people’s attention [57] and have a long and complex history of environmental engagement and activism (reviewed in [58]). There were several artists and politicians who only contributed with one or two posts about Brazilian PAs in our database, but due to their enormous number of followers, generated very high levels of public engagement. This further confirms the importance and power of celebrities in raising awareness of environmental issues, promoting science and environmental conservation [57, 58]. More generally, recent research has highlighted the existence of different personas engaged in environmental discourses on social media [59], and our results suggest a similar result for PA discussions. Exploring in greater detail which personas drive and shape PA discussions on social media can help better understand public discussions around this topic.

We found a relatively low number of georeferenced posts, possibly due to the decision to collect only Portuguese language tweets. In Brazil, the most populated states generated the most tweets. However, Iguaçu National Park, despite not being located in a highly populated state, had the highest volume for an individual PA. This supports the findings of a recent study of internet salience that found that Iguaçu National Park was the most mentioned Brazilian PA on the national and global internet [26]. This may be due to its beauty, iconic status and large annual number of visitors [51]. Iguaçu is the second most visited PA in the country, with excellent visitation structure and is located on the border between Brazil and Argentina—the second ranked country with the most geo-referenced tweets about Brazilian PAs.

Another surprising finding was the high number of urban parks georeferenced in the tweets. Urban parks are open green areas that can perform ecological, landscape and recreational functions [60]. Significantly, they are not classified as PAs according to Brazilian environmental policy. This observation highlights the importance of more effective communication regarding PAs and the need for awareness about their functions and values. Despite not being classified by the Brazilian environmental policy as PAs [3], such parks can provide important physical, social and health benefits for urban residents [61]. They are often the gateway for people’s first contact with nature, and provide opportunities to escape from the stressful pace of the city [62]. Being located in urban areas, these parks are more easily accessed when compared to other PAs and tend to have better mobile phone signal coverage. This potentially allows more real-time interaction with social media, contributing to the high volume of georeferenced postings. As an example, the Américo Renné Giannetti Municipal Park in the Minas Gerais state had the second highest volume of georeferenced posts in the entire database.

The use of social media data has recognized potential for improving knowledge and monitoring tourism interest in PAs [12, 13, 63]. Our study suggests that the most officially visited parks were also the most popular subjects for Twitter posts. Such a result matches with previous research that used different research platforms such as Google Trends [26], OpenStreetMap (OSM), and Wikipedia [64, 65] and social media platforms such as Instagram, Flickr, and Twitter [66, 67]. However, two highly-visited national parks drew attention for not being among the 30 most posted-about on Twitter: Jericoacoara National Park and the Fernando de Noronha Marine National Park. Similar discrepancies between visitation and tweets were observed in research on Nepalese Parks, due to greater local visitation and Twitter use restrictions [25]; factors that are less likely to account for the discrepancies observed in the current study. These are more likely caused by: (i) intrinsic biases in data collection. A limitation of nearly all textual content studies on the internet is the problem of synonyms [68]—alternative names or spellings for the represented entity. In the current study we used search strings that contained the full names of PAs, and may therefore have missed many tweets that used colloquial names. In the cases of Jericoacoara National Park, many Brazilians refer to the Park as "Jeri" and Fernando de Noronha Marine National Park as "Noronha"; (ii) longer names tend to be less popular on social media platforms [69] and are more likely to contain spelling errors. Abbreviations and misspellings can restrict the search for PAs, however, currently, this is the only viable and standardised way to attach a name to the PA designation at scale [26] and; (iii) Both Jericoacoara and Fernando de Noronha National Parks may have communication gaps regarding their identity as National Parks. The two parks are located in places (municipality and island, respectively) that have the same names as the Park (Jijoca de Jericoacoara municipality and Fernando de Noronha Island). This could confuse visitors or simply lead them to communicate about the place more broadly rather than the park specifically which, again, could lead to underrepresentation in our database due to our chosen search method.

The Park category was the most tweeted category, especially National Parks. The National Park has the primary objective of preserving natural ecosystems of great ecological significance and scenic beauty, enabling scientific research and the development of environmental education and interpretation activities, nature-based recreation, and ecotourism [3]. It is almost certainly that this high public interest, reflected in the substantial number of Twitter posts, is attributed to the fact that national parks incorporate recreation as a significant component of their objectives [70]. Recreation activities are strongly linked to public interest and support for PAs. Moreover, National Parks are the oldest and most visited PA category [71], and many were protected to preserve the scenic beauty of their unique landscapes along with other relevant biophysical assets [72]. In addition, in Brazil, National Parks have larger use concessions and receive more tourists, raising more resources to invest in the media. However, we noticed that a large part of our dataset contained municipal parks that are not currently present in the CNUC database. The most general explanation for this, according to government environmental analysts, is that the registration of municipal units is at the discretion of municipal environmental agencies and is not mandatory. However, registration in the CNUC is used to verify the criteria for the allocation of funds from federal public policies, such as those from federal environmental compensation. Such funds are destined exclusively for PAs recognized by CNUC as belonging to the SNUC. Our findings indicate that many parks currently unlisted by the CNUC are generating considerable value for society [73] and for biodiversity [74] and should therefore be a valid target for financial investment and improved environmental management [75].

A diversity of topics was found on tourist experiences and nature appreciation (Fig 8), corroborating the importance of PAs in bringing people closer to nature and the physical and psychological benefits they provide [11]. Unlike other content-based research on PAs, our findings did not identify topics related to iconic animals [12, 25]. Although Brazil is renowned for its megadiverse ecosystems, offering the opportunity to witness wildlife in PAs such as west indian manatee (*Trichechus manatus*), river dolphins (*Inia geoffrensis*), jaguar (*Panthera onca*), and the largest terrestrial mammal in Brazil, the tapir (*Tapirus terrestris*) [76–78], it’s important to note that many Brazilian National Parks were established with the primary goal of conserving scenic landscapes (as referenced in Article 11. [3]), rather than focusing specifically on iconic species. Furthermore, the type of social media and PA category may influence the results related to the topics that are chosen as symbols and the experiences that are chosen to be shared. Besides the topics related to tourism experiences, most of the topics that generate high levels of public interest were related to management actions and conflicts, such as fires, reduction in the size of PAs, and possible environmental crimes. This result corroborates our findings related to engagement with posts (Fig 7B). These results highlight the potential use of social media to monitor PAs in terms of cultural value generated (e.g., for tourists) or public discontent related to management decision-making [14, 79]. In the context of the spread of misinformation on Twitter, it is important to recognise that false messages may have influenced user engagement in relation to PAs, despite our observation that the presence of bots did not have a significant impact in our study (only 2.61% of the tweets were duplicates and could potentially be attributed to bots). Previous research [80] indicates that bots play a role in the dissemination of true and false information, but it is human action that amplifies false news more quickly and extensively, as they tend to arouse a greater sense of novelty and excitement in people.

It is well known that the dissemination of incorrect information can result in the delegitimisation of science, the minimisation of real threats to conservation and the generation of polarisation about the importance of protected areas. In this sense, there is a clear need for additional research that focuses on textual and behavioural content analysis in order to understand whether the issues that lead people to engage with environmental issues are false or true. These studies can shed light on the dynamics behind the spread of misinformation, as well as on effective communication strategies to combat its detrimental effects on discussions about protected areas and other environmental issues. Better understanding of human-PA interactions can also be used to inform strategies to attract societal support for conservation and PAs, to improve conservation planning and management, and to tailor communication strategies [13, 15, 37].

## Conclusions

Overall, our study adds to the rapidly growing literature on the use of culturomic metrics for monitoring human-nature interactions at large spatio-temporal scales [17]. Nevertheless, Twitter data has a few limitations that limit its suitability as a stand-alone monitoring tool. First, Twitter corpora contain high levels of slang, colloquialisms, acronyms and emoticons that can be challenging for data collection and analysis. Second, social networks are not necessarily representative of PA users or of wider society [25]. Finally, not all users enable the geolocation on their devices, limiting this type of data [42]. Thus, all social media data requires extensive cleaning and critical analysis [15]. Despite such limitations, the enormous volumes of data generated by social media platforms such as Twitter mean that their analysis can certainly generate insights into the perceptions of non-social media users [18] and, critically, allows the evaluation of macroscopic patterns of human-nature interactions [81]. Acknowledging both the limitations and potentials inherent in utilising social media as a means of investigation, we propose the integration of various online data sources, such as Wikipedia, Instagram, Facebook, and offline methodologies like questionnaires. This comprehensive approach aims to reinforce the discourse surrounding the attained outcomes and enhance the depth of analysis. Accepting the intrinsic limitations of our data, our analysis of 10 years of Twitter discourse about Brazilian PAs indicates several areas of policy that could be improved. For example, it is clear that official communication about PAs could be improved, from providing and disseminating basic information about some parks (Fig 5), to the way government agencies responsible for managing PAs communicate their decisions and campaigns (Fig 7). The lack of public engagement with conservation is often attributable to ineffective communication from scientists and decision-makers [21]. In this sense, government agencies and environmental NGOs could use data from social media to create better and more powerful awareness campaigns, potentially in partnership with celebrities and other social media ‘influencers’ (Fig 5). Furthermore, understanding online perceptions and public interest can lead to identifying topics related to conservation actions (Fig 8), where managers can pinpoint gaps and enhance strategies to maximise positive outcomes and minimise negative impacts. In a broader context, our study confirms that the use of content and engagement metrics on social media, combined with other monitoring tools such as environmental data on water quality, air, biodiversity, along with information from field monitoring and traditional media, has significant potential to enable early warnings to identify emerging conflicts in PAs (Fig 7B) and to identify public interest in these areas (Fig 7A).
